# Early detection of metastatic risk in primary cutaneous melanoma using weakly supervised learning

**DOI:** 10.1038/s41598-026-45588-w

**Published:** 2026-04-01

**Authors:** Filip Dahlén, Ivan Shujski, Filmon Yacob, Ida Häggström, Jovana Jovanovic, Olga Dudina, Ilkka Pölönen, Noora Neittaanmäki

**Affiliations:** 1 Department of Laboratory Medicine, Institute of Biomedicine, Sahlgrenska Academy, University of Gothenburg, Gothenburg, Sweden; 2 Department of Pathology, Region Västra Götaland, Norra Älvsborgs Hospital, Trollhättan, Sweden; 3 Ekkono Solutions, Varberg, Sweden; 4 Department of Electrical Engineering, Chalmers University of Technology, Gothenburg, Sweden; 5 Department of Medical Radiation Sciences, University of Gothenburg, Gothenburg, Sweden; 6 Department of Pathology, Region Västra Götaland, Södra Älvsborgs Hospital, Borås, Sweden; 7 Faculty of Information Technology, University of Jyvaskylä, Jyväskylä, Finland; 8 Department of Pathology, Region Västra Götaland, Sahlgrenska University Hospital, Gothenburg, Sweden

**Keywords:** WSI, Transformer, BioMedBERT, Melanoma, Prov-GigaPath, Foundation models, Cancer, Melanoma, Computational biology and bioinformatics, Diseases, Health care, Medical research, Computer science

## Abstract

Early identification of melanomas with high metastatic potential is crucial for treatment planning and survival prediction. We investigated whether weakly supervised WSI-based and multimodal learning approaches can identify metastatic risk in primary cutaneous melanoma beyond current clinicopathological predictors. A total of 426 routinely stained whole-slide images (WSIs), from primary melanomas (249 metastatic and 177 non-metastatic) together with corresponding clinicopathological features, were collected and divided to training and validation, and a hold-out test set. WSIs were divided into patches and encoded using Prov-GigaPath, while clinicopathological features were converted to text embeddings using BioMedBERT. We evaluated a weakly supervised transformer-based approach for slide-level metastatic risk prediction from WSIs, both alone and in combination with clinicopathological features, and compared performance to a model based on clinicopathological features alone. Both WSI-based models achieved strong performance (AUC = 0.887 and 0.883) and higher accuracy (0.847 for both) than the clinicopathological-based model (AUC = 0.849, accuracy = 0.753). This benefit was most pronounced in T2 tumors, where early risk stratification is clinically most relevant. These findings demonstrate that weakly supervised WSI-based models capture prognostically relevant information and highlight their potential for early metastatic risk stratification in primary melanoma.

**Supplementary Information:**

The online version contains supplementary material available at 10.1038/s41598-026-45588-w.

## Introduction

Cutaneous melanoma is one of the deadliest skin cancers and a growing cause of death and morbidity worldwide. The prognosis for stage III and IV melanoma patients is significantly worse compared to patients in stage I or II without metastatic disease^1^. Thus, adjuvant treatments with both targeted therapies and immunotherapy are often considered for patients in stage III and IV^2^. There is evidence indicating that metastases from a primary cutaneous melanoma often occur long before the melanoma is diagnosed^2^. However, some melanomas metastases are detected early, while some melanomas stay in a “dormant” stage for years^3^. Melanoma metastasizes lymphogenously (to regional lymph nodes or as in-transit metastases), but also hematogenously with distant metastases^1,4^. Thus, knowing if a melanoma is likely to metastasize is crucial for treatment and survival of melanoma patients.

Primary tumors contain morphological markers that may indicate metastatic potential. At the time of a primary melanoma diagnosis, it is possible to use clinico-pathological parameters to predict the likelihood of metastases^5^ and of eventual death from melanoma^6,7^.

The current prognostic biomarkers for survival in melanoma include primary tumor Breslow thickness and ulceration^6^. Although mitotic rate remains a key prognostic factor across all tumor thicknesses, it is no longer a T-category criterion in the eighth edition of the AJCC melanoma staging system. Sentinel lymph node biopsy plays a critical role in staging by detecting occult regional (stage III) nodal disease in patients with clinical stage IB-II cutaneous melanoma. Furthermore, non-nodal regional metastases (microsatellites, satellites, or in-transit metastases) are associated with adverse prognosis and classified as stage III disease. The presence of distal metastases (stage IV) further substantially worsens the prognosis^6^. However, recent studies show that more patients with stage I/IIA melanoma die than those with stage IIB–III melanoma^8,9^. With adjuvant immunotherapy now approved for stage IIB/C melanoma, it is increasingly important to identify high-risk patients within lower stages using modern risk-stratification methods.

While established prognostic markers remain central to clinical decision-making, they may fail to identify aggressive disease in some patients, motivating interest in computational approaches that can extract additional prognostic information directly from image data.

Despite the progress of self-supervised learning and foundation models in computer vision and natural language processing, their application in the medical domain remains in its early stages. One of the main reasons is the limited availability of publicly accessible data compared to other domains — a challenge particularly pronounced in pathology due to the relatively slow adoption of digital pathology and the inevitable time constraints in collecting patient data. However, recent efforts have led to the public release of several foundation models pre-trained on large-scale clinical datasets. These developments have significantly advanced computational pathology research, by lowering the barriers for smaller research groups and accelerating the translation of AI-based methods into clinical practice. Campanella et al.^10^ made a clinical benchmark of public self-supervised pathology foundation models and measured the performance of the models on two types of downstream tasks, disease detection and computational biomarkers. In general, the models trained using DINO^11^ and DINOv2^12^(SP21M, SP85M, UNI, Virchow, and Prov-GigaPath) achieved comparable performance and in biomarker prediction UNI and Prov-GigaPath were just as good or better than the other models with Prov-GigaPath performing slightly better than UNI. In biomarker prediction, an overall trend towards higher performance with larger models was also observed^10^. Recent work has further demonstrated the potential of large-scale multimodal foundation models in dermatology, with Yan et al.^13^ introducing a vision-based foundation model trained on diverse dermatological data that achieved strong performance across multiple clinical tasks, including skin disease classification and risk assessment.

In parallel, recent AI-based approaches in dermatology have explored the integration of clinical knowledge with machine learning for melanoma diagnosis. For example, Wang et al.^14^ proposed a hybrid framework combining clinical knowledge graphs with gradient-based neural networks to operationalize the Seven-Point Checklist for melanoma assessment.

Alongside diagnostic and clinically guided models, recent work has also advanced lesion segmentation using deep learning architectures, such as multiscale and dual-input networks designed for skin lesion delineation^15^. These emerging self-supervised models provide a promising starting point for further advancements in computational pathology. Alongside these developments, recent developments in computational pathology have enabled development of prognostic models based on digitized routine stained clinicopathological whole slide images (WSIs)^16^. Significant progress has been made in detecting and classifying cancers and identifying metastases in lymph nodes using machine learning models. In the domain of histological imaging, both Vision Transformers^17^ and more traditional convolutional neural network (CNN) architectures have demonstrated significant success in a range of applications, such as detecting breast cancer metastases and classifying cancer subtypes in lung, kidney and colorectal tissues^18–23^. Furthermore, transformer-based models, CNN models, and MIL models have been particularly effective in detection of metastases^21,24–28^. Despite these advancements, only a limited number of studies have focused on identifying metastatic potential in primary tumors including Brinker et al.^29^ which predicted sentinel lymph node metastatic status directly from routine histology of primary melanoma tumors using a CNN-based approach. Similarly, Knuutila et al.^30^ investigated metastatic primary cutaneous squamous cell carcinoma using a residual neural network (RNN) architecture. In another study, Kulkarni et al.^31^ analyzed primary melanoma tumors to identify patients at risk for visceral recurrence and death using a CNN combined with an RNN architecture. However, few studies have addressed early metastatic risk prediction in primary melanoma using weakly supervised approaches that avoid region-level annotations.

This study aimed to evaluate whether weakly supervised WSI-based and multimodal learning approaches can identify metastatic risk in primary cutaneous melanoma beyond current clinicopathological predictors.

## Results

The full performance metrics are summarized in Table 1. The mean ROC curves for each model are shown in Figure S1, and confusion matrices evaluated at the Youden index are presented in Figure S2.

MultiTrans, which integrates WSI and clinicopathological data, and TransMIL, based solely on WSI, both achieved an accuracy of 0.847. In comparison, BertMLP, trained on clinicopathological features alone, reached a lower accuracy of 0.753. MultiTrans yielded the highest AUC (0.887), followed by TransMIL (0.883) and BertMLP (0.849).

At the Youden operating point, MultiTrans demonstrated the highest sensitivity (0.961), correctly identifying 49 of 51 metastatic cases, followed by TransMIL (sensitivity = 0.843; 43 true positives) and BertMLP (sensitivity = 0.725; 37 true positives). In terms of specificity, TransMIL achieved the highest value (0.853), corresponding to 29 true negatives and 5 false positives. BertMLP showed intermediate specificity (0.794; 27 true negatives and 7 false positives), while MultiTrans exhibited lower specificity (0.676; 23 true negatives and 11 false positives). Subgroup analyses based on tumor Breslow thickness are represented in Table 2. In tumor stage T2 TransMIL and MultiTrans performed significantly better compared to BertMLP. A more comprehensive ablation study is available in supplementary materials.

**Table 1:** Performance metrics for MultiTrans, TransMIL and BertMLP.

Model	AUC	Accuracy	Sensitivity	Specificity	TP	FP	FN	TN
MultiTrans	**0.887**	**0.847**	**0.961**	0.676	**49**	11	**2**	23
TransMIL	0.883	**0.847**	0.843	**0.853**	43	**5**	8	**29**
BertMLP	0.849	0.753	0.725	0.794	37	7	14	27

**Table 2:** Subgroup analysis on hold-out test set for MultiTrans, TransMIL and BertMLP. WSI’s were divided into stages. T1: Breslow thickness ≤ 1 mm. T2: 1 mm < Breslow thickness ≤ 2 mm. T3: 2 mm < Breslow thickness ≤ 4 mm. T4: Breslow thickness > 4 mm. Statistical significance of accuracy differences between models was assessed using paired bootstrap resampling within Breslow thickness subgroups.

Tumor thickness	Metastatic WSI	Non-metastatic WSI	Accuracy (met vs. non-met)			*p*–value	
			MultiTrans	TransMIL	BertMLP	MultiTrans vs. BertMLP	TransMIL vs. BertMLP
T1	0	9	1.00 vs. 1.00	1.00 vs. 1.00	1.00 vs. 1.00	ns	ns
T2	11	15	0.91 vs. 0.67	0.56 vs. 1.00	0.09 vs. 0.93	0.002	0.003
T3	8	8	0.88 vs. 0.50	0.75 vs. 0.63	0.50 vs. 0.50	ns	ns
T4	32	2	1.00 vs. 0.00	0.97 vs. 0.00	1.00 vs. 0.00	ns	ns
T all	51	34	0.961 vs. 0.676	0.843 vs. 0.853	0.753 vs. 0.794	ns	0.0176

The contribution of clinicopathological features was evaluated by training the BertMLP and MultiTrans models using different subsets of clinical variables (Table 3). For BertMLP, the highest AUC was observed when using Breslow thickness alone (AUC = 0.849), with incremental decreases in performance as additional features were included. Models trained using only ulceration or mitotic rate showed substantially lower performance (AUC = 0.691 and 0.608, respectively). When all clinical features were included, the BertMLP achieved an AUC of 0.802. In contrast to BertMLP, MultiTrans performance was largely unaffected by variations in the included clinicopathological features. For a more comprehensive sensitivity analysis of clinicopathological parameters, see Supplementary material.

**Table 3:** AUC values for the BertMLP and MultiTrans models trained using different subsets of clinicopathological features.

Clinicopathological features	AUC	
	MultiTrans	BertMLP
All	**0.887**	0.802
Breslow, Ulceration & Mitoses	0.869	0.819
Breslow & Ulceration	**0.887**	0.809
Breslow & Mitoses	0.884	0.843
Breslow	0.874	**0.849**
Ulceration	0.884	0.691
Mitoses	0.879	0.608
Diameter	0.880	0.675
Regression	0.855	0.627

**Fig. 3:**
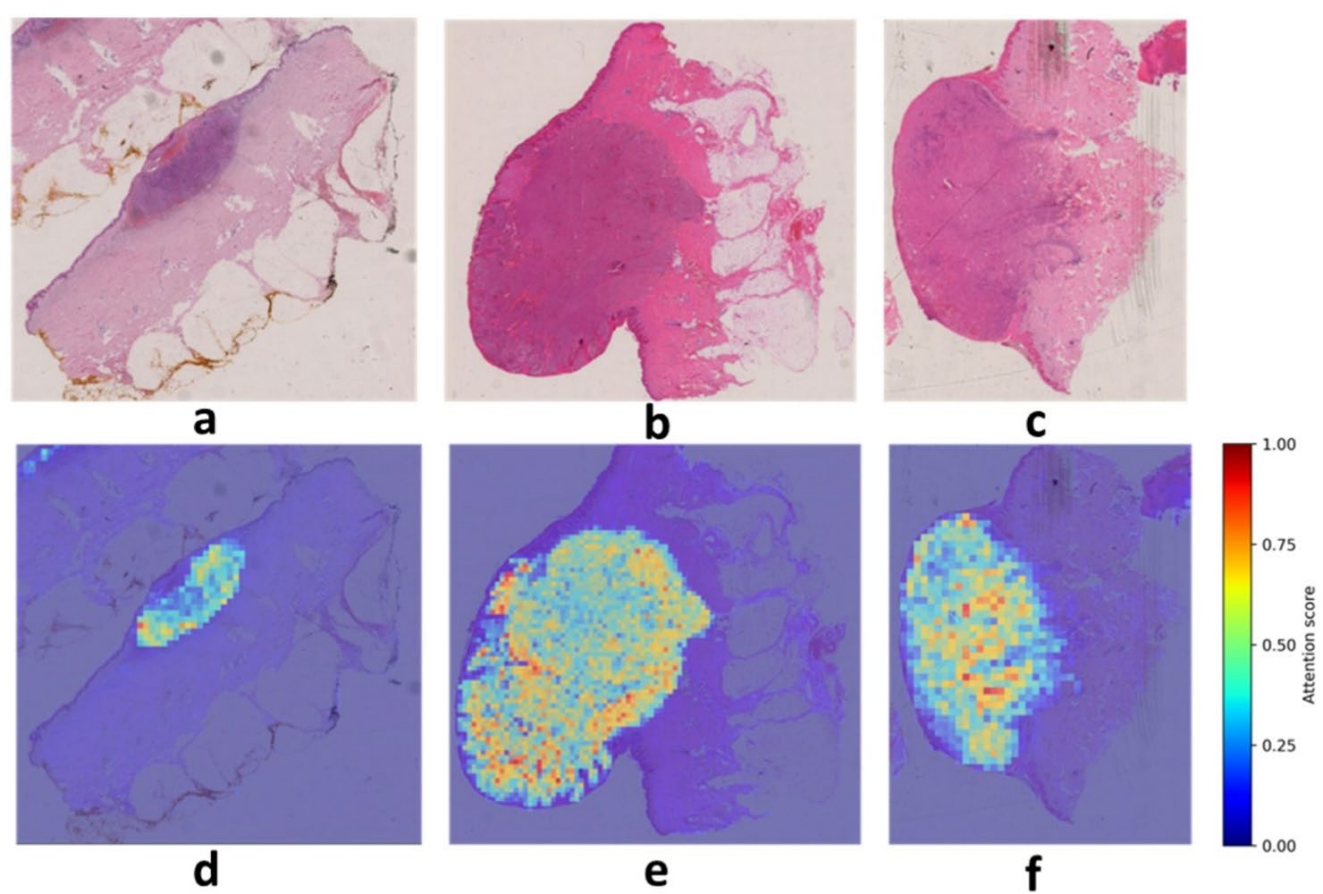
Visualization of class activation maps derived from all attention weights generated by MultiTrans over the original WSI (**d**, **e** and **f**) and the corresponding H&E image (**a**, **b** and **c**). Sample **a**,** d** represents a non-metastatic primary melanoma with a diameter of 7 mm and a Breslow thickness of 1.7 mm. It shows ulceration, dermal mitosis, and no regression. Sample **b**,** e** represents a metastatic primary melanoma with a diameter of 16 mm and a Breslow thickness of 11 mm. It shows ulceration, dermal mitoses, and no regression. Sample **c**,** f** represents a metastatic primary melanoma with a diameter of 10 mm and a Breslow thickness of 6.5 mm. It shows ulceration, dermal mitoses, and no regression.

To further interpret model predictions, attention heatmaps were used to visualize the spatial distribution of model attention across entire WSIs. Figure 3 shows representative heatmaps for three correctly classified cases, illustrating how MultiTrans attends to multiple regions across the slide when forming slide-level predictions.

### Expert review of model selected regions

To complement the heatmap analysis and identify recurring morphological patterns across cases, a separate model-guided tile clustering analysis was performed using only the highest-attention regions pooled across true-positive cases. Qualitative assessment of TransMIL-selected tiles by an expert dermatopathologist revealed clear differences between metastatic and non-metastatic cases.

### Metastatic cases

**Fig. 4:**
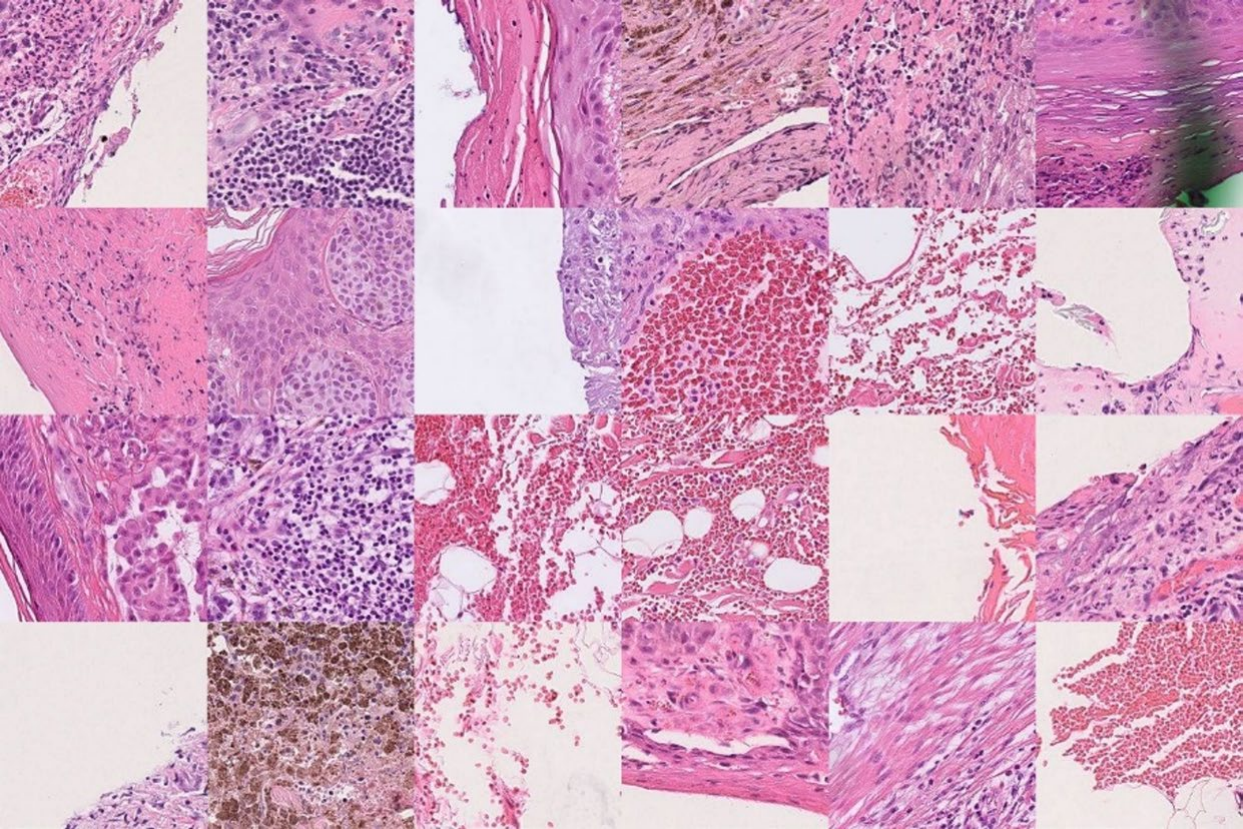
Guided clustering of high-attention tiles from true-positive metastatic cases classified by the TransMIL model. Representative image tiles from the cluster with the highest WSI coverage are shown. The displayed cluster represents the most consistently attended morphological pattern across metastatic cases.

In correctly classified metastatic (true positive) cases, most highlighted tiles corresponded to vascular and ulceration-associated features, and inflammatory infiltrates in the dermis Fig. 4. Interestingly, most of the high-attention regions were located outside the regions containing tumor cells while only a few showed incohesive tumor islands, and isolated tumor cells located outside the main tumor mass in the dermis.

In correctly classified cases that developed metastases at later time points during follow-up, all high-attention regions were consistently located outside the main tumor mass. These regions predominantly correspond to vascular structures, including blood vessels and occasional lymphatic vessels, as well as ulceration-associated areas. No intravascular tumor growth was identified on H&E slides. Some regions represented nonspecific stromal or adipose tissue, and none corresponded to tumor cell–dense regions, Fig S3.

### Non-metastatic cases

**Fig. 5:**
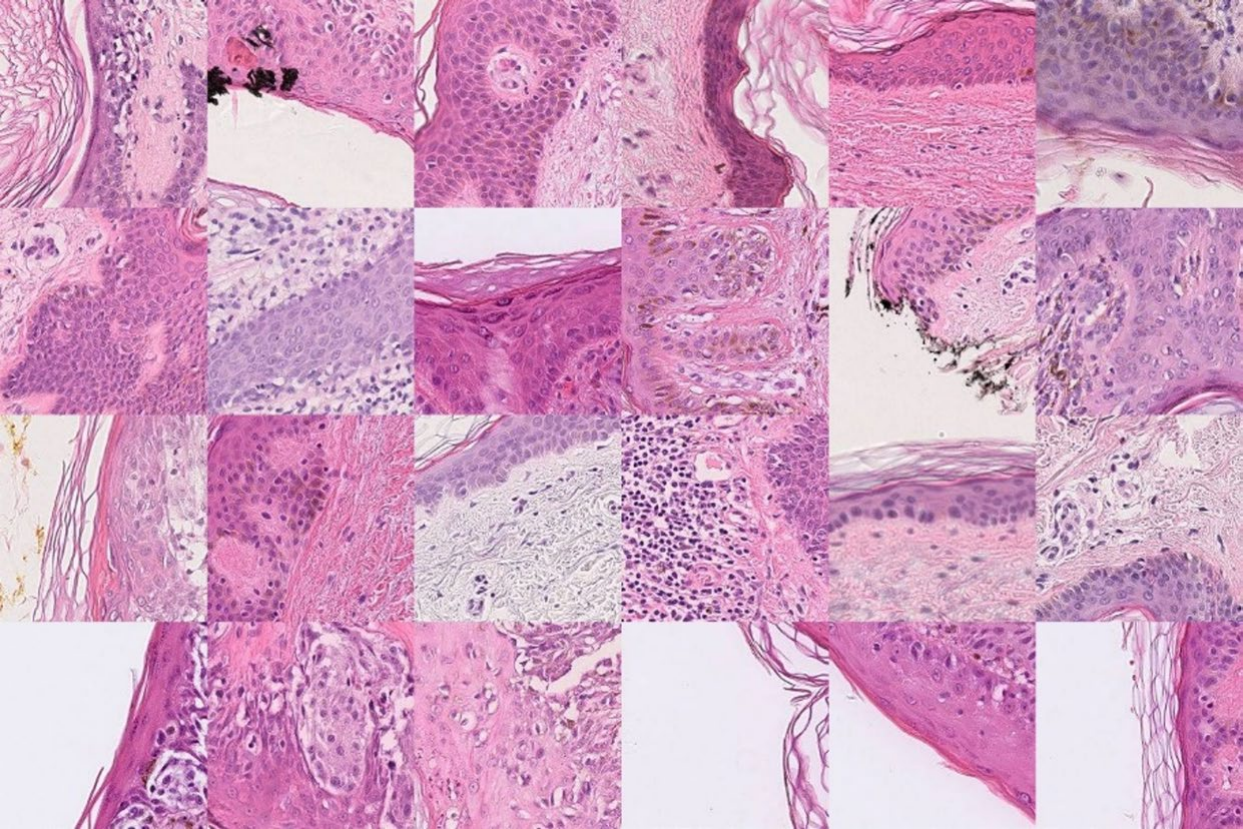
Guided clustering of high-attention tiles from true-negative non-metastatic cases. Representative image tiles from the cluster with the highest WSI coverage are shown. The displayed cluster represents the most consistently attended morphological pattern across non-metastatic cases.

In contrast, tiles highlighted in correctly classified non-metastatic (true negative) cases predominantly corresponded to epidermal structures, Fig. 5. These mostly included intact non-ulcerated epidermis with or without an in-situ melanoma component while some attention maps highlighted keratin layer without signs of ulceration. Some TN regions showed epidermis and underlying superficial inflammation, but completely lacked attention to dermal tumor cells, vascular structures or ulceration.

### Misclassified cases

Misclassified cases were characterized primarily by non-specific dermal regions, fat and tissue-processing artifacts, Figures S4–S5.

## Discussion

In this study, we investigated the potential of weakly supervised WSI-based and multimodal analysis, applied to routine histopathology without region-level annotations, for early identification of metastatic risk in primary cutaneous melanoma. By comparing WSI-based models with a clinicopathological features baseline, we aimed to assess whether WSI-based modeling can detect metastatic risk beyond what is achievable using current clinicopathological standards. The results showed that TransMIL and MultiTrans demonstrated superior decision-level performance compared with BertMLP, highlighting the added value of image-based modeling for metastatic risk prediction.

Stratified analysis by Breslow thickness (Table 2) revealed marked differences in model behavior across clinically relevant subgroups. In the T1 subgroup (Breslow thickness ≤ 1 mm), no metastatic cases were present, and all models correctly classified all samples. In T2 subgroup (Breslow thickness 1–2 mm), both TransMIL and MultiTrans outperformed BertMLP in terms of accuracy (*p* = 0.003 and *p* = 0.002, respectively). MultiTrans achieved the most balanced performance, correctly classifying 10 out of the 11 metastatic cases and 10 out of the 15 non-metastatic cases. In contrast, BertMLP showed very limited ability to identify metastatic cases in this subgroup (accuracy = 0.09), despite high accuracy for non-metastatic cases, indicating a strong bias toward over-predicting non-metastatic disease. TransMIL favored detection of non-metastatic cases by correctly classifying all non-metastatic cases but at the expense of reduced accuracy for metastatic cases.

In the T3 subgroup (Breslow thickness 2–4 mm), TransMIL demonstrated the most balanced performance across metastatic and non-metastatic cases, whereas BertMLP performed at chance level. MultiTrans maintained higher accuracy for metastatic cases but continued to misclassify a substantial proportion of non-metastatic cases. In the T4 subgroup (> 4 mm), which was dominated by metastatic cases, all models correctly identified nearly all metastatic samples, but consistently misclassified the two non-metastatic cases, reflecting the strong association between tumor thickness and metastatic risk.

Overall, these results indicate that TransMIL achieved the most consistent and balanced performance across Breslow thickness strata, while BertMLP performed poorly, particularly in T2 and T3 tumors where early risk stratification is clinically most relevant. MultiTrans consistently prioritized sensitivity, leading to increased detection of metastatic cases but reduced specificity. These findings underscore the limitations of models based on clinicopathological features for early metastatic risk prediction and highlight the value of WSI-based approaches, maintaining balanced performance across tumor thicknesses.

Qualitative analysis of model-selected regions revealed consistent differences between metastatic and non-metastatic cases. In metastatic correctly classified cases, TransMIL model predominantly attended to vascular structures, ulceration-associated regions, microenvironmental features including inflammatory infiltrates whereas tumor cells received minimal model attention. Importantly, this attention pattern was also observed in tumors that developed metastases at later time points and classified as lower AJCC stages (stage II) at diagnosis. This suggests that microenvironmental cues associated with metastatic risk may be present well before clinical manifestation and before histopathologically detected clues for metastases (intravasal growth). In contrast, non-metastatic regions were primarily characterized by attention to intact, non-ulcerated epidermis and in situ melanoma components. False-positive (FP) and false-negative (FN) cases were enriched for non-specific stromal tissue and specimen borders, and tissue-processing artifacts, with limited presence of vascular or ulceration-related features, indicating that misclassification often occurs when informative microenvironmental signals are weak or ambiguous.

These observations are consistent with established mechanisms of melanoma metastasis, which typically proceed through hematogenous and lymphatic dissemination, and further underscore the prognostic significance of vascular and ulceration-associated microenvironments. Together, the concordance between model attention patterns and known pathological processes supports the biological plausibility of weakly supervised WSI-based models for early metastatic risk stratification in primary melanoma.

Several prior studies have explored the prediction of metastatic risk from routine histopathology in cutaneous malignancies, using a range of machine learning paradigms and supervision strategies. Brinker et al.^29^ predicted sentinel lymph node metastasis directly from WSIs of primary melanoma tumors, reporting an AUC of 0.618 when using WSIs alone, 0.616 when using clinicopathological features alone, and 0.613 when combining both modalities. Knuutila et al.^30^ investigated metastatic primary cutaneous squamous cell carcinoma and achieved AUCs of 0.747 using WSIs alone and 0.804 using clinicopathological features, with a further increase to 0.917 when incorporating AI-based predictions together with clinical risk factors in a risk factor model. Kulkarni et al.^31^ focused on predicting visceral recurrence and disease-specific mortality from primary melanoma WSIs using a CNN–RNN architecture, reporting AUCs of 0.905 and 0.880 in two independent validation cohorts.

Substantial methodological differences exist between these studies and the present work, which may explain the differences in model performances. First, the clinical endpoints differ across studies. While Kulkarni et al.^31^ predicted long-term outcomes such as visceral recurrence and death, our study aims to predict early metastatic potential. This difference in prediction target may inherently affect achievable model performance and partially account for variability in reported AUCs.

Second, prior studies relied on varying degrees of manual annotation and patch-level supervision. Brinker et al.^29^ and Knuutila et al.^30^ explicitly delineated tumor regions and assigned patch-level labels before training convolutional neural networks. Although such approaches can improve signal-to-noise ratios, they may also introduce potential sources of bias and label noise due to inter-observer variability and predefined assumptions regarding which regions are informative. In contrast, the present study employs a weakly supervised learning paradigm in which no region-level or patch-level annotations are required. Instead, all image patches from each WSI contribute to slide-level predictions, allowing the model to learn discriminative patterns through self-attention mechanisms. This approach enables the capture of both local and global contextual information across entire WSIs and may facilitate the identification of subtle morphological cues not explicitly annotated in conventional workflows.

Third, none of the studies utilized a foundation model for feature extraction. In prior work, convolutional networks were typically trained from scratch or initialized using ImageNet pretraining, which may limit their ability to capture complex morphological structures. In contrast, our approach decouples feature extraction from classification by leveraging a pretrained foundation model to generate robust patch-level embeddings, which are subsequently aggregated using a transformer-based architecture. This design likely improves generalizability and reduces overfitting, particularly in settings with limited sample sizes, and may contribute substantially to the observed performance gains.

Fourth, variations in dataset size may also contribute to differences in reported performances across studies. Brinker et al.^29^ analyzed 415 H&E slides from primary melanoma tumors with known sentinel lymph node status, whereas Knuutila et al.^30^ relied on a smaller cohort of 104 H&E slides from primary cutaneous squamous cell carcinoma. Kulkarni et al.^31^ used 108 patients for training and 155 patients for testing. Differences in cohort size and composition can influence model stability, confidence intervals, and susceptibility to overfitting, which likely contributes to the observed variability in AUC values across studies. Furthermore, differences in multimodal integration strategies may also influence predictive performance. Knuutila et al.^30^ demonstrated that combining image-based predictions with clinical risk factors can markedly improve discrimination. In the present study, WSIs alone already provided strong predictive performance, with only a modest additional benefit from incorporating clinicopathological features. This finding suggests that the learned image representations encode much of the prognostically relevant information, although it may also indicate that the applied data fusion strategy did not optimally align image embeddings with structured features. More advanced multimodal fusion or joint embedding approaches may further enhance performance and warrant investigation in future work. Taken together, these findings suggest that differences in reported AUC values across studies, including the performance observed in the present work, are likely attributable to a combination of factors such as differences in clinical endpoints, cohort size and composition, supervision strategies, the use of foundation model–based feature extraction, and transformer-based attention mechanisms. Importantly, our results demonstrate that high predictive performance can be achieved without extensive manual annotations or handcrafted risk factor models, highlighting the potential scalability and clinical applicability of weakly supervised, foundation model–driven approaches in computational pathology.

Interestingly, a recent study by Lallas et al.^32^ used deep learning on dermatoscopic images of melanomas and demonstrated a similar performance to Breslow thickness and ulceration in predicting metastatic potential. More recently, Yan et al.^13^ demonstrated that a large-scale dermoscopic foundation model (PanDerm) can predict melanoma metastasis and long-term outcomes beyond standard clinical risk factors, further supporting the prognostic value of image-based representations. Furthermore, Amaral et al.^33^ evaluated a model integrating clinicopathologic factors with gene expression profiling (CP-GEP) to predict disease recurrence in stage I/II cutaneous melanoma and concluded that CP-GEP effectively identifies patients at high risk of recurrence, particularly in stage I/IIA disease. Our current multimodal approach combined WSIs and clinicopathological features encoded as text embeddings. Incorporating dermoscopy images and gene expression profiles could represent additional layers of multimodality, potentially improving clinical relevance and model performance.

In our study, all three models could identify 7 of 8 melanoma patients, initially classified as non-metastatic but who developed metastases during the follow up period. Among the seven correctly classified cases, four developed metastases within one year. All four tumors had Breslow thicknesses above the average for metastatic cases (6.5, 6.5, 6.0, and 8.0; mean = 4.9), exhibited ulceration, showed mitotic activity, and displayed no evidence of regression. The remaining three cases developed metastases after two years. These tumors had Breslow thicknesses of 6.0, 5.1, and 5.5; only one exhibited ulceration, while all showed mitotic activity and no regression. The eighth case, which was incorrectly classified as non-metastatic, developed metastases after five years. This tumor had a Breslow thickness of 1.8, below the non-metastatic average, showed no ulceration or regression, but exhibited mitotic activity.

We included tumor diameter, mitotic rate, and regression as exploratory predictors of metastatic potential. Permutation-based sensitivity analyses (Supplementary Materials) showed that randomization of tumor diameter and regression resulted in modest changes in model performance, indicating limited but consistent contributions within the multivariable framework. In contrast, randomization of mitotic rate led to a larger and more variable reduction in performance, suggesting that this feature captures information related to tumor proliferative activity that is relevant for metastatic risk prediction, despite not being part of current AJCC staging. Consistent with established clinical knowledge, perturbation of Breslow thickness and ulceration produced the most pronounced performance decreases. Complementary feature-ablation experiments based on model retraining (Table 3) further supported these findings, showing that Breslow thickness alone yielded the strongest performance for the clinical-only BertMLP model, while the multimodal framework remained robust to variations in clinical feature composition.

The primary contribution of this work lies in its clinical application and learning paradigm rather than in proposing a novel fusion architecture. Specifically, the novelty of the study is the use of weakly supervised multimodal learning to enable early identification of metastatic risk in primary cutaneous melanoma using routine histopathology, without reliance on region-level annotations or handcrafted risk models.

While no pixel-level tumor annotations were used to quantitatively validate attention maps, this is consistent with the weakly supervised learning paradigm, which intentionally avoids reliance on region-level ground truth. The attention mechanisms are designed to highlight regions contributing to slide-level predictions rather than to perform explicit tumor segmentation. Qualitative review of attention heatmaps by an expert pathologist confirmed that highlighted regions corresponded to histologically relevant tumor areas.

Evidence suggests that melanoma metastases often occur many months before a primary melanoma diagnosis is made^3^ which makes it crucial to identify these aggressive tumors in an early stage. Metastases to regional lymph nodes via lymphatics is the most common form of spread, with around 50% of those who develop metastases having nodal disease as their first site of clinically-detected recurrence^1,4^. However, metastases in a distant organ with no evidence of previous or current lymph node disease is seen in about 30% of those who develop metastatic melanoma^34,35^ suggesting dissemination of malignant cells exclusively via the bloodstream. Lymph node metastases are often diagnosed earlier than metastases at distant sites, with a median interval of 16 months between primary diagnosis and the detection of nodal metastases in one study^34^, while distant metastases tend to be detected a median of 25–40 months after primary diagnosis^36^. In our study of 426 tumors, 249 were metastatic, and of those 128 were in stage III showing lymph node or local in transit metastases while 121 showed distal metastases (stage IV). Despite the minimum of three year follow up and sentinel node biopsies conducted in all the patients in the non-metastatic group, some of the non-metastatic group may have developed undetected micrometastatic disease. Furthermore, occasionally melanomas show very late clinical appearance of metastases, sometimes more than 10 years after the primary melanoma was excised^37^.

One limitation of this study is the dataset size. Although a cohort of 426 WSIs is valuable, it may not capture the full variability of tumor morphology and metastatic behavior present in larger, more diverse populations, potentially limiting generalizability. The dataset size plays a critical role in improving model performance^38^, and simultaneous scaling of dataset and model size has been shown to yield substantial performance gains. Another limitation is that disease-specific survival was not predicted. Additionally, not all WSIs had complete clinicopathological feature annotations, which likely reduced model performance. Each WSI also contained an identification number written on the glass slide at scanning, which could potentially influence predictions; however, inspection of attention maps revealed no focus on these regions. Two sets of clinical text inputs were evaluated for BertMLP—one including gender and age and one excluding them, the latter representing the final approach. Incorporation of gender and age resulted in lower performance, contrasting with findings reported by Mervic et al.^37^. One possible explanation is dataset imbalance, with male patients overrepresented, potentially introducing bias; however, this warrants further investigation.

A further limitation is that model development and evaluation were conducted using data from a single institution, and no external validation cohort was available. Consequently, the generalizability of the proposed approach across institutions, scanners, and staining protocols could not be fully assessed. Nevertheless, weakly supervised learning combined with foundation model–based feature extraction may help mitigate overfitting to institution-specific characteristics by promoting more generalizable histomorphological representations. Future work will focus on validation using external, multi-institutional cohorts and publicly available datasets to further assess robustness and clinical applicability.

In conclusion, TransMIL trained solely on WSI was able to detect early signs of metastatic potential in primary melanomas with high accuracy, outperforming BertMLP trained on clinicopathological parameters that represent the current prognostic standard. This benefit was most pronounced in T2 tumors, where early risk stratification is clinically most relevant. MultiTrans, which combines image data with clinicopathological text embeddings, achieved similar performance to TransMIL. These findings demonstrate that weakly supervised WSI-based models capture prognostically relevant information and highlight their potential for early metastatic risk stratification in primary melanoma. Predicting early signs of metastatic potential from primary tumors may enable targeted treatment strategies for patients at high risk of disease progression.

### Methods

The Swedish Ethical Review Authority (Dnr 2023–06786−02) approved this study. Since all the material was anonymized, consent to participate was waived by the Swedish Ethical Review Authority. The research was conducted in accordance with the tenets of the Declaration of Helsinki.

**Fig. 6:**
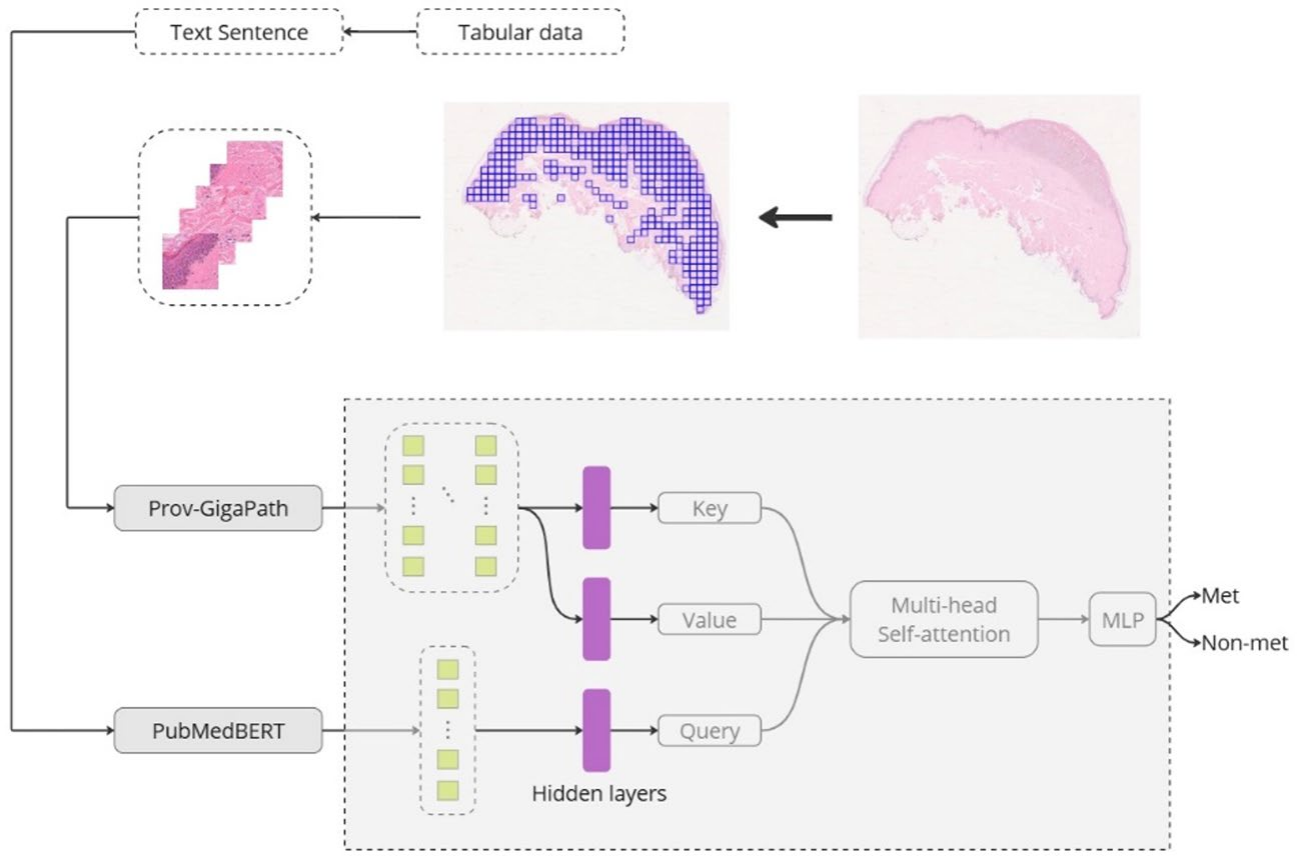
Method overview. The WSI is first tiled into patches, with each patch converted into a feature embedding using Prov-GigaPath. For each corresponding set of clinical data, a text sentence is constructed, and text embeddings are generated using BioMedBERT. The image embeddings and text embeddings are then projected into queries, keys, and values through three linear layers. Attention is computed between the image and text embeddings, and the aggregated output is classified using an MLP layer.

An overview of the method is shown in Fig. 6.

### Dataset

The data was retrospectively collected at the Sahlgrenska University Hospital between 2016 and 2023. Inclusion criteria were a primary cutaneous malignant melanoma stage IB-IV with information about possible metastases and in case of non-metastatic tumor, a negative sentinel node examination and a minimum of 3 years follow-up time. The exclusion criteria were patient cases with metastatic malignant melanoma having more than one primary malignant melanoma tumor to avoid uncertainty of which was the primary tumor that metastasized. One glass slide per tumor harboring the largest Breslow thickness was collected and scanned unidentified using a scanner NanoZoomer S360 Hamamatsu at 40X magnification. The complete dataset consisted of 426 WSIs representing 426 primary melanomas (249 metastatic and 177 non-metastatic) from 425 patients (one patient had two non-metastatic melanomas), detailed in Table 2. The size of the WSI ranged from 1.4GB up to 5.8GB with dimensions between (157 440 × 55 296) and up to (215 040 × 109824) pixels. The total size of the dataset was around 1.4 TB.

**Table 4:** Distribution of clinical and clinicopathological features across metastatic and non-metastatic classes. Breslow thickness and diameter are presented as mean (min - max) values in millimeters. Mitoses, ulceration, and regression are reported as the fraction of occurrences (present/not present) within each class.

	All included			Training and validation set			Hold-out test set		
	Total	Met	Non-met	Total	Met	Non-met	Total	Met	Non-met
**Slides**									
WSI	426	249	177	341	198	143	85	51	34
Patches	2 838 864	1 659 336	1 179 528	2 272 424	1 319 472	952 952	566 440	339 864	226 576
**Gender**									
Male	231	138	93	182	107	75	49	31	18
Female	195	111	84	159	91	68	36	20	16
**Age (Mean**,** IQR)**									
Male	66 (29–92)	66 (32–92)	65 (29–90)	65 (29–92)	65 (32–92)	65 (29–89)	68 (42–90)	68 (43–89)	67 (42–90)
Female	65 (17–94)	67 (21–94)	63 (17–92)	65 (17–94)	67 (21–94)	63 (17–92)	64 (27–94)	64 (27–94)	65 (32–86)
**Stage**									
IB	-	-	108	-	-	88	-	-	20
IIA	-	-	37	-	-	29	-	-	8
IIB	-	-	21	-	-	16	-	-	5
IIC	-	-	11	-	-	10	-	-	1
III	-	46	-	-	27	-	-	19	-
IIIA	-	8	-	-	6	-	-	2	-
IIIB	-	8	-	-	6	-	-	2	-
IIIC	-	16	-	-	15	-	-	1	-
IV	-	121	-	-	94	-	-	27	-
**clinicopathological features**									
Breslow (mean, std, p-value)	3.7 (0.3–35)	4.9 (0.5–35)	1.9 (0.3–9.5)	3.5 (0.5–20)	4.6 (0.5–20)	1.9 (0.8–9.5)	4.9 (0.3–35)	6.3 (1.1–35)	1.8 (0.3–0.7)
p-value	$\:{1.70\cdot\:10}_{}^{-23}$								
Diameter (mean, std)	14.3 (3.0–80.0)	16.3 (3.5–80)	11.6 (3.0–59)	14.1 (3.0–59)	16.0 (4.0–45)	11.6 (3.0–59)	14.9 (3.5–80)	17.7 (3.5–80)	10.9 (4.0–23)
p-value	$\:{7.43\cdot\:10}_{}^{-9}$								
Dermal Mitoses (count)	417	245 (59%)	172 (41%)	332	195 (59%)	137 (41%)	85	50 (59%)	35 (41%)
p-value	$\:{3.56\cdot\:10}_{}^{-12}$								
Ulceration (count)	426	248 (58%)	178 (42%)	341	198 (58%)	143 (41%)	85	50 (59%)	35 (41%)
p-value	$\:{4.45\cdot\:10}_{}^{-6}$								
Regression (count)	404	240 (59%)	164 (41%)	322	192 (60%)	130 (40%)	82	48 (59%)	34 (41%)
p-value	$\:{1.74\cdot\:10}_{}^{-4}$								
Non-metastatic patients who developed metastases during follow-up (proportion, count)	-	19%, *n* = 48	-	-	20%, *n* = 40	-	-	14%, *n* = 8	-
**Time (Mean**,** Median**,** IQR)**									
Time between primary melanoma diagnosis and detection of metastases	-	4.5 months, 0 months, (0–96 months)	-	-	4.7 months, 0 months, (0–96 months)	-	-	3.5 months, 0 months, (0–60 months)	-
Time between primary melanoma diagnosis and detection of metastases for patients who were initially non-metastatic and developed metastases during follow-up	-	23 months, 12 months, (12–96)		-	23 months, 12 months, (12–96)	-	-	22 months, 12 months, (12–60)	-
Follow up time	-	-	64 months, 65 months, (37–86)	-	-	65 months, 65 months, (37–86)	-	-	62 months, 62 months, (42–82)

### Clinicopathological features

Baseline clinicopathological characteristics are summarized in Table 4. Metastatic melanomas exhibited significantly greater Breslow thickness and more frequent ulceration compared with non-metastatic tumors across all dataset splits (*p* < 0.001). In addition, dermal mitoses, ulceration, regression, and larger primary tumor diameter were more frequently observed in metastatic cases (*p* < 0.001). Among patients initially classified as non-metastatic, 19% in the full cohort developed metastases during follow-up (20% in the training/validation set and 14% in the hold-out test set). The median time from diagnosis to detection of metastases was 23 months (range: 12–96 months) in the full cohort, with comparable values across dataset splits. When considering all patients with metastatic tumors, the median time from diagnosis to metastasis detection was 4.5 months (range: 0–96 years). Median follow-up time was 64 months (interquartile range: 37–86 months) for the overall cohort, with similar follow-up durations observed in the training/validation and hold-out test sets, ensuring adequate longitudinal assessment of disease progression. None of the patients received systemic therapy during the follow-up period prior to the occurrence of the metastases.

### Statistical analysis

The Mann–Whitney U test was used to compare each clinicopathological parameter between metastatic and non-metastatic groups. The test was applied separately to each parameter to evaluate whether their value distributions differed between groups. Corresponding p-values are reported in.

To assess whether performance differences between models were statistically significant, pairwise comparisons were performed using DeLong’s test for ROC AUC and paired bootstrap tests for accuracy-based metrics at the Youden index. In addition, sensitivity analyses were performed using both permutation-based perturbation of individual clinicopathological features and feature-ablation experiments in which models were retrained using different subsets of histopathological features. Further methodological details are provided in the Supplementary Methods.

### Model-guided tile selection and clustering analysis

To interpret model predictions, two complementary attention-based analyses were performed.

First, attention heatmaps were generated to visualize the spatial distribution of model attention across entire whole-slide images (WSIs), highlighting regions contributing to slide-level predictions.

Second, a model-guided tile selection and clustering analysis was conducted to identify recurring high-attention morphological patterns across the true positive, true negative, false positive, false negative and true positive cases that later developed metastases. For each WSI in each case, the top 20 tiles receiving the highest attention scores from the trained TransMIL model were extracted and pooled into a single set of model-selected regions.

To identify shared patterns across cases, tile embeddings from all pooled regions were ℓ2-normalized and clustered using k-means clustering. Clusters were ranked according to WSI coverage, defined as the number of unique WSIs contributing at least one tile to a given cluster, thereby prioritizing patterns consistently present across multiple slides rather than patterns driven by a single slide. For visualization and expert review, representative tiles were selected based on proximity to the cluster centroid, while enforcing diversity across WSIs such that at most one tile per WSI was selected before additional tiles were included. The cluster with the highest WSI coverage was selected for detailed analysis, as it represented the most common attention-associated tissue pattern across metastatic cases.

### Image feature extraction

The WSIs were tiled into 224 by 224 patches at 10X magnification using OpenSlide^39^. There was no overlap between the patches and only patches with at least 15% tissue were kept for further analysis. In total, 2.3 million patches were generated for the training set and 0.5 million patches were generated for the test set. After processing WSIs into patches, features were extracted using the whole-slide model Prov-GigaPath^40^. Prov-GigaPath is a foundation model designed for analyzing gigapixel pathology slides by extracting slide-level embeddings for diverse clinical applications. It uses a two-stage approach with a tile encoder, pretrained using DINOv2^12^, to capture local features from image tiles, and a slide encoder, leveraging masked autoencoder pretraining with LongNet^41^, to model global features across the entire slide. Prov-GigaPath was pretrained on the dataset Prov-Path comprising over 1.38 billion tiles from 171,189 pathology slides, representing 31 tissue types and data from over 30,000 patients.

### Clinical feature extraction

Textual descriptions were generated from the tabular clinicopathological data presented in Table 2. Each description followed a consistent sentence structure and was generated using two templates: (1) a template used for MultiTrans that included all available clinicopathological features, and (2) a simplified template used for BertMLP that included only Breslow thickness. When analyzing the effect of different clinicopathological features, the textual descriptions were modified to include only the relevant feature(s) while preserving the same sentence structure; an example template is shown below (3).

1. *“Whole slide image of malignant melanoma*, has a breslow thickness of * mm and a diameter of * mm, shows *, *, *”.
2. *“Whole slide image of malignant melanoma*, has a breslow thickness of * mm.”
3. *“Whole slide image malignant melanoma*,* shows mitotic activity.”*

Here, * represents values extracted from the tabular data. For example, one generated sentence reads:

“*Whole slide image of malignant melanoma*,* has a breslow thickness of 1.2 mm and a diameter of 13.0 mm*,* shows mitotic activity*,* regression*,* and ulceration.”*

Text embeddings were then generated for each sentence using the pre-trained large language model BioMedBERT^42^. In cases where tabular data were missing, the corresponding information was simply omitted from the sentence, as BioMedBERT will generate embeddings of a fixed size regardless of input length.

### Models

#### MultiTrans

MultiTrans incorporates a trainable layer that projects the text embeddings into Queries, while the image embeddings are projected into Keys and Values. These representations are then processed through Multi-Head Attention, producing attended features that are aggregated and passed through a final MLP layer for classification.

#### TransMIL

The TransMIL as described by Shao et al.^15^ was used. The TransMil model, given a bag of image embeddings, will perform multi-head self-attention^43^ on the input embeddings to capture the correlation between the different embeddings. Furthermore, the attended features are fed to a Pyramid Position Encoding Generator (PPEG) that will encode the spatial information before being fed to the last Transformer layer to aggregate the morphological information. Finally, the aggregated features are fed to a single MLP layer for classification.

#### BertMLP

Classification of text embeddings was performed using a MLP consisting of three layers with ReLU activation functions in between.

### Training the models

Each model was trained on 341 samples using five-fold cross validation, with splits stratified by the class label. In each fold, 80% of the data was allocated for training and 20% for validation. A final model was obtained through majority voting across the five cross-validation models, and its performance was evaluated on an independent holdout test set consisting of 85 samples. The same hyperparameters were used for all models, with four attention heads in the Multi-Head Attention mechanism for both TransMIL and MultiTrans. Training was conducted with a learning rate of 5E^*−* 5^, for 50 epochs, with early stopping applied after 10 epochs of no improvement. Optimization was performed using the Adam optimizer with a weight decay of 10E *−* 3. One bag (batch) was fed to the model at a time. Training was performed on a single GPU on a DGX A100 system and, on average, converged within 10 min for both TransMIL and MultiTrans.

## Supplementary Information

Below is the link to the electronic supplementary material.

Supplementary Material 1

## Data Availability

The datasets generated and/or analysed during the current study will be available at the melanoma dataset via AIDA (10.23698/aida/melmet). The code is available on GitHub (https://github.com/DermPathology-AI/multimodal-melanoma).

## References

[1] Gershenwald, J. et al. Melanoma staging: Evidence-based changes in the american joint committee on cancer eighth edition cancer staging manual. *CA Cancer J. Clin.* **67**, 472–492 (2017). 29028110 10.3322/caac.21409 PMC5978683

[2] Thompson, J. & Williams, G. When does a melanoma metastasize? implications for management. *Oncotarget* **15**, 374–378 (2024). 38870033 10.18632/oncotarget.28591 PMC11174830

[3] Ossowski, L. & Aguirre-Ghiso, J. Dormancy of metastatic melanoma. *Pigment Cell. Melanoma Res.* **23**, 41–56 (2010). 19843243 10.1111/j.1755-148X.2009.00647.x PMC2821074

[4] Adler, N., Haydon, A., McLean, C., Kelly, J. & Mar, V. Metastatic pathways in patients with cutaneous melanoma. *Pigment Cell. Melanoma Res.* **30**, 13–27 (2017). 27900851 10.1111/pcmr.12544

[5] Huang, H., Fu, Z., Ji, J., Huang, J. & Long, X. Predictive values of pathological and clinical risk factors for positivity of sentinel lymph node biopsy in thin melanoma: A systematic review and meta-analysis. *Front. Oncol.* **12**, 817510 (2022). 35155254 10.3389/fonc.2022.817510 PMC8829564

[6] Balch, C. et al. Prognostic factors analysis of 17,600 melanoma patients: validation of the american joint committee on cancer melanoma staging system. *J. Clin. Oncol.* **19**, 3622–3634 (2001). 11504744 10.1200/JCO.2001.19.16.3622

[7] Dillekås, H., Rogers, M. S. & Straume, O. Are 90% of deaths from cancer caused by metastases? *Cancer Med.* **8**, 5574–5576 (2019). 31397113 10.1002/cam4.2474 PMC6745820

[8] Landow, S. M., Gjelsvik, A. & Weinstock, M. A. Mortality burden and prognosis of thin melanomas overall and by subcategory of thickness, SEER registry data, 1992–2013. *J. Am. Acad. Dermatol.* **76**, 258–263 (2017). 27887797 10.1016/j.jaad.2016.10.018

[9] Whiteman, D. C., Baade, P. D. & Olsen, C. M. More people die from thin melanomas (1 mm) than from thick melanomas (> 4 mm) in Queensland, Australia. *J. Invest. Dermatol.* **135**, 1190–1193 (2015). 25330295 10.1038/jid.2014.452

[10] Campanella, G. et al. A clinical benchmark of public self-supervised pathology foundation models. *arXiv* **2407**, 06508 (2024). 10.1038/s41467-025-58796-1 PMC12003829 40240324

[11] Zhang, H. et al. DINO: DETR with improved denoising anchor boxes for end-to-end object detection. (2022). arXiv 2203.03605.

[12] Oquab, M. et al. DINOv2: learning robust visual features without supervision. *arXiv* **2304**, 07193 (2024).

[13] Yan, S. et al. A multimodal vision foundation model for clinical dermatology. *Nat. Med.* **31**, 2691–2702 (2025). 40481209 10.1038/s41591-025-03747-y PMC12353815

[14] Wang, Y. et al. Integrating Clinical Knowledge Graphs and Gradient-Based Neural Systems for Enhanced Melanoma Diagnosis via the Seven-Point Checklist. *IEEE Trans. Neural Networks Learn. Syst.* (2025). 10.1109/TNNLS.2025.3600443 40907050

[15] Li, X. et al. MSDUNet: A Model based on Feature Multi-Scale and Dual-input Dynamic Enhancement for Skin Lesion Segmentation. *IEEE Trans. Med. Imaging* (2025). 10.1109/TMI.2025.3549011 40053626

[16] Acs, B., Rantalainen, M. & Hartman, J. Artificial intelligence as the next step towards precision pathology. *J. Intern. Med.* **288**, 62–68 (2020). 32128929 10.1111/joim.13030

[17] Dosovitskiy, A. et al. An image is worth 16×16 words: transformers for image recognition at scale. arXiv 2010.11929 (2021).

[18] Shao, Z. et al. TransMIL: transformer-based correlated multiple instance learning for whole-slide image classification. *Adv. Neural Inf. Process. Syst.* **34**, 2136–2147 (2021).

[19] Zeid, M. A. E., El-Bahnasy, K. & Abo-Youssef S. E. Multiclass colorectal cancer histology image classification using vision transformers. In Proc. ICICIS, 224–230 (2021).

[20] Yacob, S. J. V. K. et al. Weakly supervised detection and classification of basal cell carcinoma using graph transformers on whole-slide images. *Sci. Rep.* **13**, 33863 (2023). 10.1038/s41598-023-33863-z PMC10169852 37160953

[21] Waqas, M., Ahmed, S. U., Tahir, M. A., Wu, J. & Qureshi, R. Exploring multiple instance learning: a brief survey. *Expert Syst. Appl.* **250**, 123893 (2024).

[22] De Logu, F. et al. Recognition of cutaneous melanoma on digitized histopathological slides via artificial intelligence algorithms. *Front. Oncol.* **10**, 1559 (2020). 33014803 10.3389/fonc.2020.01559 PMC7508308

[23] Hekler, A. et al. Pathologist-level classification of histopathological melanoma images with deep neural networks. *Eur. J. Cancer*. **115**, 79–83 (2019). 31129383 10.1016/j.ejca.2019.04.021

[24] Sun, D. et al. Artificial intelligence-based pathological application to predict regional lymph node metastasis in papillary thyroid cancer. *Curr. Probl. Cancer*. **53**, 101150 (2024). 39342815 10.1016/j.currproblcancer.2024.101150

[25] Tan, L. H. Y. et al. Colorectal cancer lymph node metastasis prediction with weakly supervised transformer-based multiple instance learning. *Med. Biol. Eng. Comput.* (2023). 10.1007/s11517-023-02799-x PMC10182132 36809427

[26] Lu, M. Y. et al. Data-efficient and weakly supervised computational pathology on whole-slide images. *Nat. Biomed. Eng.* **5**, 555–570 (2021). 33649564 10.1038/s41551-020-00682-w PMC8711640

[27] Jansen, P. et al. Deep learning detection of melanoma metastases in lymph nodes. *Eur. J. Cancer*. **188**, 161–170 (2023). 37257277 10.1016/j.ejca.2023.04.023

[28] Siarov, J. et al. Deep learning model shows pathologist-level detection of sentinel node metastasis of melanoma and intranodal nevi on whole-slide images. *Front. Med.* **11**, 1418013 (2024). 10.3389/fmed.2024.1418013 PMC11374739 39238597

[29] Brinker, T. J. et al. Deep learning approach to predict sentinel lymph node status directly from routine histology of primary melanoma tumours. *Eur. J. Cancer*. **154**, 227–234 (2021). 34298373 10.1016/j.ejca.2021.05.026

[30] Knuutila, R. P. et al. Identification of metastatic primary cutaneous squamous cell carcinoma using artificial intelligence analysis of whole-slide images. *Sci. Rep.* **12**, 13696 (2022). 35701439 10.1038/s41598-022-13696-y PMC9197840

[31] Kulkarni, P. M. et al. Deep learning based on standard H&E images of primary melanoma tumors identifies patients at risk for visceral recurrence and death. *Clin. Cancer Res.* **26**, 1126–1134 (2020). 31636101 10.1158/1078-0432.CCR-19-1495 PMC8142811

[32] Lallas, K. et al. Prediction of melanoma metastasis using dermatoscopy deep features: an international multicentre cohort study. *Br. J. Dermatol.* **191**, 847–848 (2024). 38992891 10.1093/bjd/ljae281

[33] Amaral, T. et al. Identification of stage I/II melanoma patients at high risk for recurrence using a model combining clinicopathologic factors with gene expression profiling. *Eur. J. Cancer*. **182**, 155–162 (2023). 36739215 10.1016/j.ejca.2022.12.021

[34] Meier, F. et al. Metastatic pathways and time courses in the orderly progression of cutaneous melanoma. *Br. J. Dermatol.* **147**, 62–70 (2002). 12100186 10.1046/j.1365-2133.2002.04867.x

[35] Leiter, U., Meier, F., Schittek, B. & Garbe, C. The natural course of cutaneous melanoma. *J. Surg. Oncol.* **86**, 172–178 (2004). 15221923 10.1002/jso.20079

[36] Mervic, L. Time course and pattern of metastasis of cutaneous melanoma differ between men and women. *PLoS One*. **7**, e32955 (2012). 22412958 10.1371/journal.pone.0032955 PMC3295777

[37] Schmid-Wendtner, M. H. et al. Late metastases of cutaneous melanoma: an analysis of 31 patients. *J. Am. Acad. Dermatol.* **43**, 605–609 (2000). 11004614 10.1067/mjd.2000.107234

[38] Zhai, X., Kolesnikov, A., Houlsby, N. & Beyer, L. Scaling vision transformers. (2022). arXiv 2106.04560.

[39] Goode, A., Gilbert, B. & Heller, J. OpenSlide: a vendor-neutral software foundation for digital pathology. *J. Pathol. Inf.* **4**, 27 (2013). 10.4103/2153-3539.119005 PMC3815078 24244884

[40] Xu, H. et al. A whole-slide foundation model for digital pathology from real-world data. *Nature* (2024). 10.1038/s41586-024-07441-w PMC11153137 38778098

[41] Ding, J. et al. LongNet: scaling transformers to 1,000,000,000 tokens. (2023). arXiv 2307.02486.

[42] Gu, Y. et al. Domain-specific language model pretraining for biomedical natural language processing. (2020). arXiv 2007.15779.

[43] Vaswani, A. et al. Attention is all you need. arXiv 1706, 03762 (2017).

