# Supplementary material for: Early detection of metastatic risk in primary cutaneous melanoma using weakly supervised learning
Source: Sci Rep. 2026 Apr 1;16:11234. doi: 10.1038/s41598-026-45588-w (PMC13046822; doi:10.1038/s41598-026-45588-w)
Supplement: Supplementary file 1 — Supplementary Material 1 [file 41598_2026_45588_MOESM1_ESM.docx]

**Supplementary Materials**


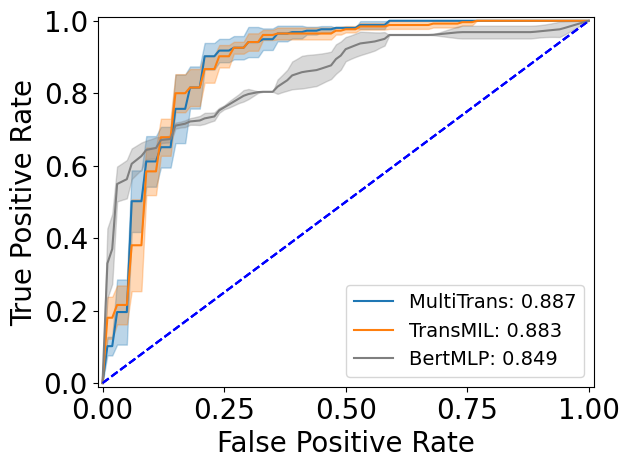


**Figure S1**: Mean receiver operating characteristic curves with noted AUC of MultiTrans, TransMIL and BertMLP from the five-fold cross validation tested on the hold-out test set (n=85).


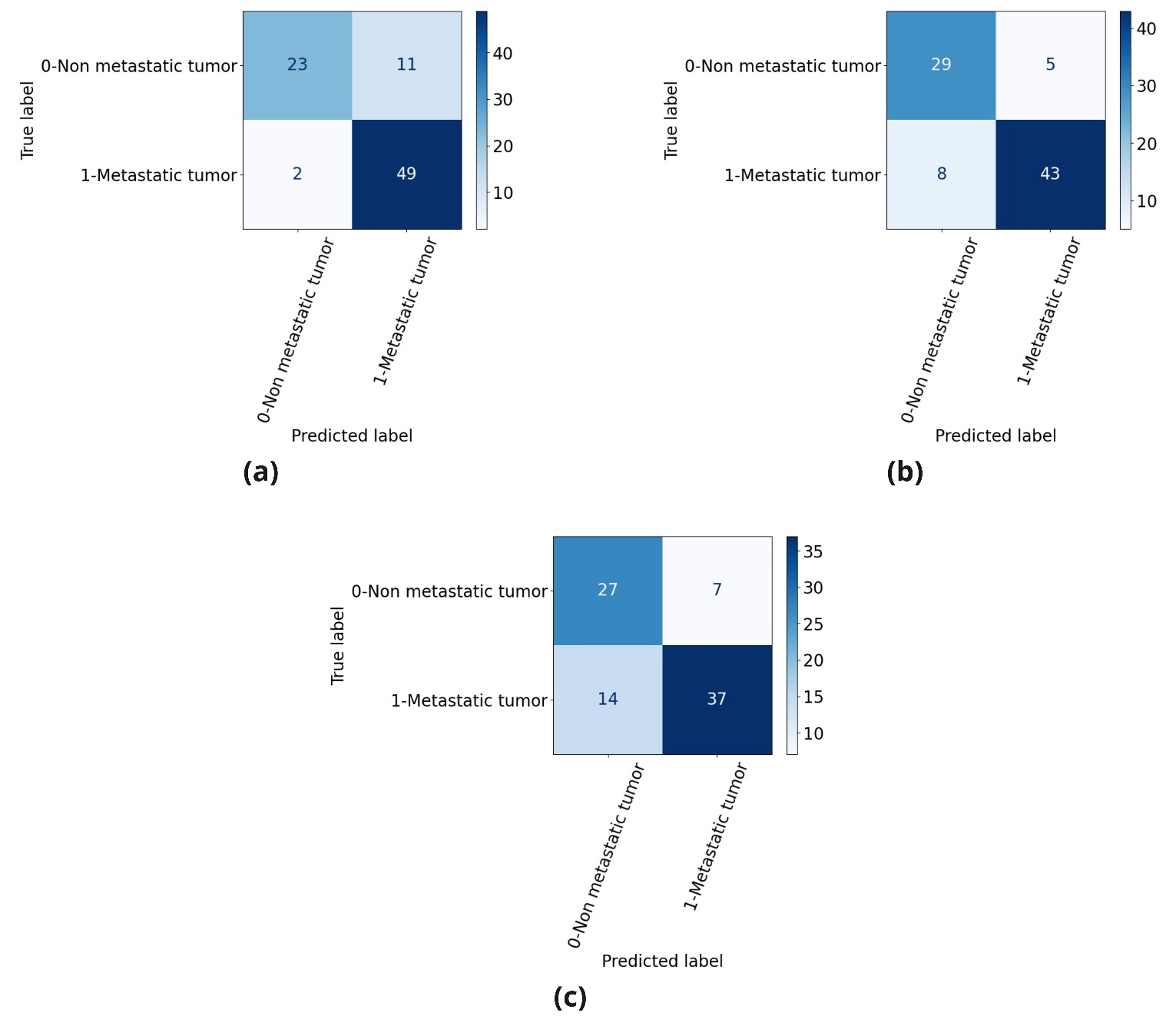


**Figure S2**: Confusion Matrices evaluated at the Youden Index for (a) MultiTrans, (b) TransMIL and (c) BertMLP from the five-fold cross validation tested on the hold-out test set (n=85).

***Statistical Analysis***

For the comparison between MultiTrans and TransMIL, neither DeLong’s test nor the paired bootstrap test indicated a statistically significant difference (DeLong: p = 0.7095; bootstrap: p = 0.4652), indicating comparable ranking and decision-level performance between the two models. When comparing MultiTrans with BertMLP, no statistically significant difference in ROC AUC was observed (DeLong: p = 0.0877), nor was a significant difference detected in the paired bootstrap test (p = 0.232). For the comparison between TransMIL and BertMLP, the difference in ROC AUC did not reach statistical significance (DeLong: p = 0.3084); however, the bootstrap test revealed a statistically significant difference in accuracy-based performance (p = 0.0176). Overall, these results show that differences between models are more evident at the decision threshold than in global ranking performance, highlighting the value of reporting both threshold-independent and threshold-dependent metrics.

***Sensitivity Analysis of Clinicopathological Features***

To evaluate the contribution and robustness of individual clinicopathological features, complementary sensitivity analyses were performed using both permutation-based perturbation and feature ablation through model retraining.

For permutation-based sensitivity analysis, values of individual clinical variables were independently randomized by resampling from the empirical distribution of the test set, while all remaining variables were kept unchanged. Following permutation, clinical embeddings were regenerated for each case, resulting in 100 independently permuted embeddings per test case for each variable. These embeddings were evaluated using the BertMLP classifier trained on non-permuted data. Model performance was assessed using the area under the receiver operating characteristic curve (AUC), and summary statistics were reported across permutations.

For feature-ablation experiments, the BertMLP and MultiTrans models were retrained using different subsets of clinicopathological features, including single-feature and multi-feature combinations. Model performance was evaluated using the same training, validation, and testing protocol as in the main experiments, and AUC values were reported for each feature subset.

***Permutation-based sensitivity analysis***

The BertMLP classifier achieved an AUC of 0.802 when evaluated on non-permuted clinical embeddings. Sensitivity analyses were first performed for exploratory clinicopathological features. Randomization of tumor diameter resulted in a modest decrease in predictive performance, with a mean AUC of 0.790 and a standard deviation of 0.011 across permutations. Randomization of mitotic rate led to a more pronounced reduction in performance (mean AUC = 0.731, standard deviation = 0.032), whereas randomization of regression had a limited effect on model performance (mean AUC = 0.811, standard deviation = 0.010).

Sensitivity analyses were subsequently conducted for established prognostic features. Randomization of Breslow thickness resulted in a substantial decrease in performance (mean AUC = 0.736, standard deviation = 0.021), while randomization of ulceration led to a further reduction (mean AUC = 0.665, standard deviation = 0.050). These results indicate that perturbation of established prognostic variables has a larger impact on model performance than perturbation of exploratory features.


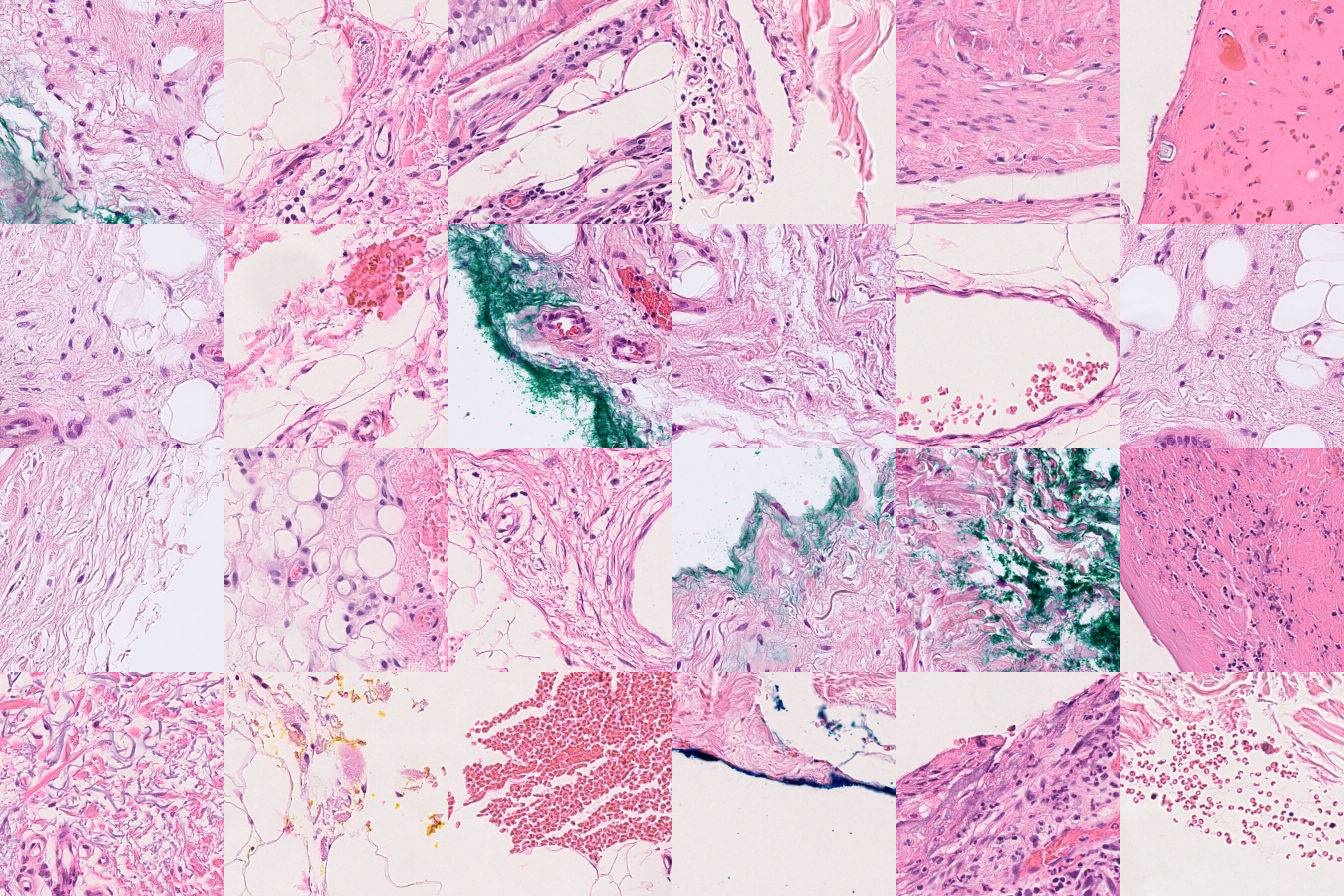


**Figure S3**. Guided clustering of high-attention tiles from true-positive cases that later developed metastases. Representative image tiles from the cluster with the highest WSI coverage are shown. The displayed cluster represents the most consistently attended morphological pattern across the correctly classified cases that later developed metastases.


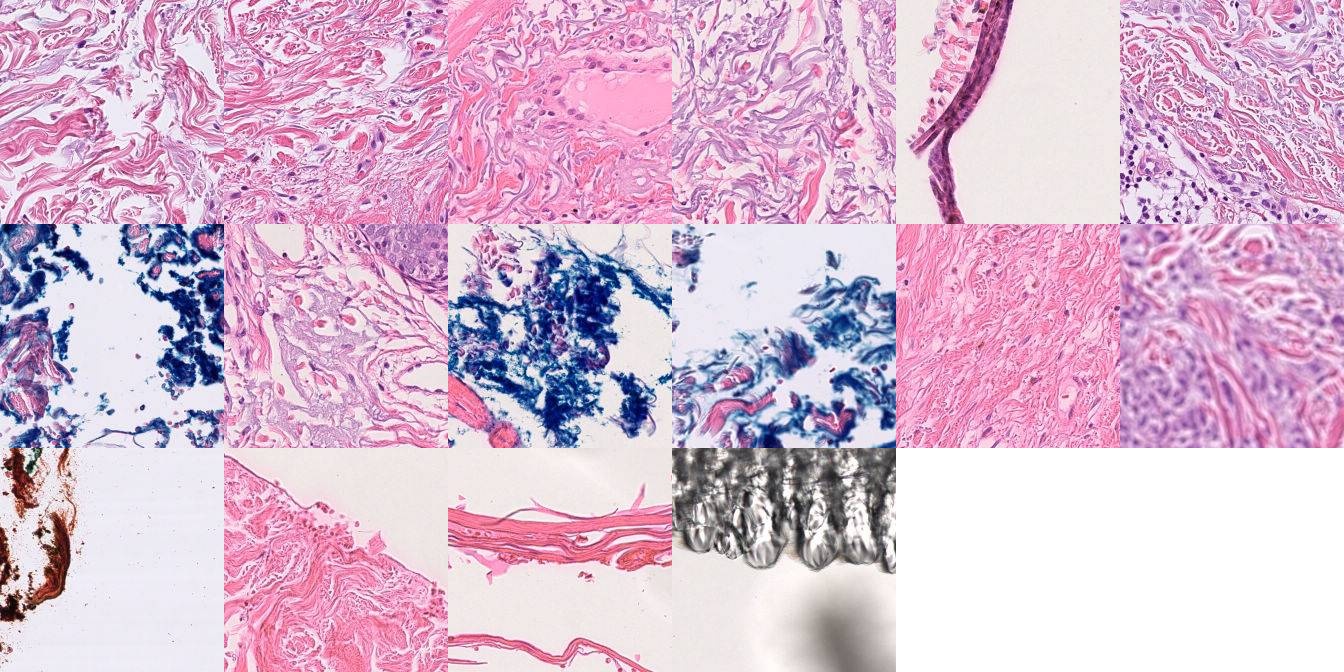


**Figure S4**. Guided clustering of high-attention tiles from false-negative cases. Representative image tiles from the cluster with the highest WSI coverage are shown. The displayed cluster represents the most consistently attended morphological pattern across the incorrectly classified metastatic cases.


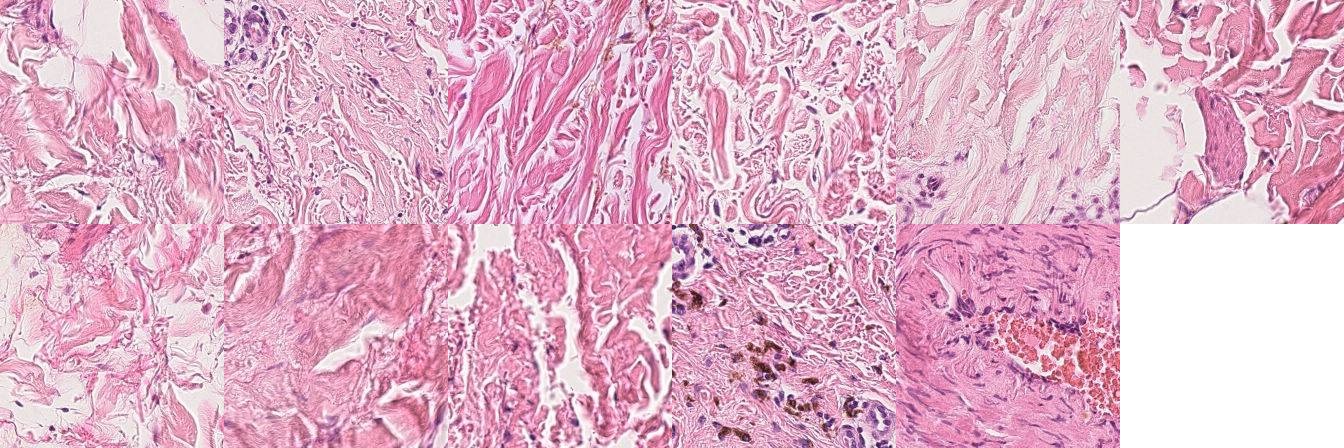


**Figure S5**. Guided clustering of high-attention tiles from false-positive cases. Representative image tiles from the cluster with the highest WSI coverage are shown. The displayed cluster represents the most consistently attended morphological pattern across the incorrectly classified non-metastatic cases.
